# Prospective validation and real-time implementation of an automated machine learning postoperative mortality prediction model

**DOI:** 10.1016/j.bja.2025.11.042

**Published:** 2026-01-17

**Authors:** Theodora Wingert, Tiffany Williams, Briana Syed, Brian Hill, Tristan Grogan, Andrew Young, Zarah Antongiorgi, Valiollah Salari, Alexandre Joosten, Ira Hofer, Eran Halperin, Maxime Cannesson, Eilon Gabel

**Affiliations:** 1 Department of Anesthesiology & Perioperative Medicine, University of California Los Angeles, Los Angeles, CA, USA; 2 University of Texas Medical Branch, John Sealy School of Medicine, Galveston, TX, USA; 3 Age Bold, Inc., Los Angeles, CA, USA; 4 Department of Medicine Statistics Core, David Geffen School of Medicine at UCLA, University of California Los Angeles, Los Angeles, CA, USA; 5 Department of Anesthesiology, Perioperative & Pain Medicine, Icahn School of Medicine at Mount Sinai, New York, NY, USA; 6 Department of Computer Science, University of California Los Angeles, Los Angeles, CA, USA

**Keywords:** implementation, machine learning, mortality, postoperative outcomes, prospective validation, risk prediction

## Abstract

**Background:**

Machine learning prediction models require prospective validation to ensure implementation fidelity and feasibility. Our primary objective was to prospectively validate a previously reported postoperative mortality prediction model in inpatients undergoing surgery. Our secondary objective was to evaluate feasibility of a pilot clinical decision support tool.

**Methods:**

We prospectively validated and implemented a random forest machine learning model trained to predict in-hospital mortality using data from a single academic medical centre. A reduced 32-feature model was implemented into the electronic health record (EHR) using a real-time data mart at the same institution. To assess model performance, the area under the receiver operating characteristic curve (AUROC), area under the curve precision-recall (AUCPR), and other performance measures were calculated. To assess feasibility, implementation workflow metrics were evaluated and a survey was administered to anaesthesiologists trained to use the pilot clinical decision support tool.

**Results:**

The AUROC for the prospectively implemented model was 0.874 (95% confidence interval [CI] 0.860—0.887), and the AUCPR was 0.111. By comparison, the AUROC for the 58-feature model was 0.925 (95% CI 0.900—0.947), and for ASA physical status the AUROC was 0.814 (95% CI 0.802—0.827) and the AUCPR was 0.103. The implementation demonstrated feasibility through real-time data updates, automated transfer of model outputs to the EHR, and provider survey entries.

**Conclusions:**

This prospective validation and EHR implementation of a previously published random forest machine learning model predicting postoperative in-hospital mortality demonstrated acceptable real-world performance of the implemented model and feasibility of integrating such a system into clinical practice.

Despite an abundance of interest in artificial intelligence (AI) and machine learning (ML) in medicine, much of the focus remains on generation of new models rather than getting such models into the hands of providers.^1-5^ Validation and implementation pathways for AI/ML in real-world settings are a much-needed step to ensure reliability, generalisability, and clinical utility.^6,7^ There are currently very few examples of ML-based prediction models of postoperative mortality that have been validated prospectively and implemented in an automated fashion.^8^

We previously reported development of a high-performing ML model for predicting postoperative mortality using preoperative clinical features.^9^ This model was based on readily-extractable features and performed better than ASA physical status, Charleson Comorbidity Index, and other preoperative scores on most measures of model performance. Perioperative risk prediction tools, particularly those available in real time, have the potential to guide decision-making about appropriate care locations, equipment and monitoring needs to support safe care, counselling of families, and clinical assessment, particularly in high-acuity or time-limited settings.^1^ Several validation studies outside of the perioperative space, have highlighted the challenges inherent in external validation of implemented prediction tools, such as the Epic Sepsis Model or digital early warning scores.^10,11^ Prospective validation and real-world implementation are essential to ensure these tools translate from high-performing models to tools that meaningfully improve patient outcomes.

There are many technical and practical hurdles to prospective validation of an AI/ML model within a large-scale medical system, requiring automated electronic health record (EHR) data validation, AI-enabled servers, and robust systems for data security and high-throughput efficiency.^3^ All of these elements must occur within a short enough timeframe for model outputs to be clinically relevant. Few institutions have demonstrated feasibility and prospective validation of automated delivery of perioperative prediction models, particularly in the context of mortality prediction.^8^

Therefore, the primary objective of this study was to prospectively validate a previously reported mortality prediction model in inpatients undergoing surgery. The secondary objective was to evaluate the feasibility of a pilot clinical decision support (CDS) tool.

## Methods

This was a single-centre study designed to assess both prospective validation and implementation feasibility of a postoperative mortality prediction model in inpatients at an academic medical centre (UCLA). Although prospective model validation was designated as the primary aim and feasibility as the secondary aim, the two were inherently interdependent, as successful implementation was a necessary prerequisite for prospective evaluation. The study was Institutional Review Board (IRB)-exempt (UCLA IRB#15-0518) and adheres to STROBE and TRIPOD-AI guidelines.^12^

### Data source

The UCLA Perioperative Data Warehouse automatically validates and integrates laboratory, medication, billing, and clinical documentation data from the EHR (Epic Systems, Madison, WI, USA) and external sources (e.g. echocardiogram data-bases).^13^ For clinical model variables, project-specific packages were executed every 6 h to extract data more frequently into the real-time data mart. A minimally processed list of admitted surgical patients was retrieved directly from EHR tables every 15 min. Together, these two data input pathways facilitated implementation, with overall data latency determined primarily by the 6-h extraction schedule. Variables used in the implemented model are listed in Supplementary Table 1.

### Model features and study population for the model

The previously published model was trained and tested on both inpatients and outpatients undergoing surgery at one of two UCLA medical centres (Ronald Reagan UCLA Medical Center [RRUMC] and Santa Monica Medical Center) or six outpatient surgery centres from 2013 to 2018.^9^ The binary outcome for the classification models was in-hospital mortality, defined using EHR notes and structured data elements. The random forest model generated a risk score from 0 to 1, and included 58 structured clinical features (e.g. age, vitals, laboratory results, surgical details), intentionally excluding ASA physical status to avoid reliance on clinician interpretation. Full details are available in the original publication.^9^

### Model features and study population for prospective implementation

The implemented model used 32 features, compared with 58 in the original model. A reduction in the total number of variables was necessary, as each individual variable required integration into a real-time data mart with limited capacity and processing constraints (Supplementary Fig. 1). Twenty-four timestamps with minimal impact on model output were removed. Procedure code and admission case number were omitted because these were not available until after the surgery. Presurgical location was omitted because of data quality concerns, as the ‘perioperative’ location was occasionally assigned to patients who had been transferred from a ward or ICU, leading to potential misclassification. It is also significant that the implementation cohort included only inpatients age ≥18 yr at RRUMC, the larger and higher acuity medical centre. To reduce potential bias and statistical impact of repeat cases in this cohort with possible intervention, only the first surgical encounter per patient was included in the analysis. However, in the original model, individual patients who underwent multiple surgeries were all included in the training and test datasets, accounting for an additional difference between the original model and the implemented model.

### Prospective implementation period and population

The 32-feature model was implemented, with automated model output delivered to the EHR from May 2021 to December 2023 (2 yr, 8 months). Implementation was limited to RRUMC inpatients in order to: (1) target the high-acuity inpatients and (2) allow for a targeted provider cohort for the planned CDS tool.

### Model deployment architecture and data flow

To enable deployment, several data processing steps were implemented. Inpatients at RRUMC were identified in real-time every 15 min via Application Programming Interface (API) using Fast Healthcare Interoperability Resources (FHIR), which transmitted key data directly from EHR tables to AI-enabled servers. The 32 model features were validated through the Perioperative Data Warehouse and transmitted for scheduled inpatients every 6 h. The model ran automatically upon data receipt using Python (version 3.11; Python Software Foundation, Wilmington, DE, USA) in Jupyter Note-book (version 6.4.5; Project Jupyter) on specialised servers co-ordinated with the UCLA Office of Health Informatics and Analytics to ensure performance and data governance (further details and Supplementary Fig. 1 with Database Structure and Pathways).

Model output with numeric value >0.5 was transmitted back to the EHR via Health Level Seven (HL7) international messaging standards as a custom field, ‘High Risk for Mortality’. A threshold of 0.5 was selected for two main reasons: (1) Youden’s index for our model was 0.43 and (2) we found that 0.5 yielded a clinically manageable number of daily patients. The model was trained to generate an output value from 0 to 1, with higher output predicted to have higher probability of mortality. The model output was not created as to be a direct estimate of probability (i.e. a model output >0.5 does not correspond to a 50% chance of mortality).

A dynamic patient list labelled ‘High Risk for Mortality Surgical Add-on’ was created within the EHR, automatically updating to display high-risk patients for clinician review. The list of patients booked for surgery was able to be updated at 15-min intervals, as the data input required minimal data processing from EHR tables. In contrast, clinical variables model inputs were updated every 6 h via the real-time data mart because of the more complex data joins and automated validation steps required to ensure accuracy and integrity of these variables. The random forest model was automatically repeatedly executed in Python, with data transfer of the output triggered only when the new output numeric value differed from the prior value.

To ensure system reliability, automated logging, redundancy, and error checking mechanisms were in place with alerts for any data or processing failures. Multiple outputs were often logged per patient; for this analysis, the most recent model output before surgery was used.

### Pilot clinical decision support tool

The CDS tool was a passive intervention that was active from March 2022 to December 2023 (1 yr, 9 months). Patients with a model output >0.5 appeared on a real-time patient list visible to clinicians. At RRUMC, the float anaesthesiologist, who was responsible for inpatient add-on procedures, recovery, and airway support, was asked to review the ‘High Risk for Mortality Surgical Add-on’ list during their shifts Monday through Friday 07:00 to 17:00. About 10 anaesthesiologists rotated in this role and were engaged before and throughout implementation to ensure workflow alignment.

The patient list was not intended to direct or alter patient care, but to highlight potentially high-risk inpatients scheduled for surgery, enabling timely recognition and evaluation by clinicians. The tool provided automated risk stratification to inform, but not dictate, clinical decision-making.

### Clinical decision support feasibility

To evaluate CDS feasibility and clinician engagement, a voluntary passive post-intervention survey was made available to anaesthesiology providers involved in assessment of flagged patients. This survey was passively available to clinicians within the EHR by an embedded case-specific link. Upon accessing the case-specific survey, clinician respondents were presented with a three question form using Qualtrics^®^ (Qualtrics, Provo, UT, USA) with the full survey available in the Supplementary Material. Survey responses were subsequently joined with other clinical data for analysis. All data collection and storage procedures were Health Insurance Portability and Accountability Act (HIPAA) -compliant and conducted in accordance with institutional data governance policies. Respondents were asked ‘Were any preoperative interventions undertaken for this patient?’ Providers were then asked to ‘Select any/all interventions taken for the patient’.

We performed exploratory analyses to examine whether provider interventions in response to the model meaningfully influenced clinical outcomes (Supplementary Tables 2 and 3). Sample sizes were very small for these, so they are purely exploratory. We conducted subgroup analysis of cases during the CDS period who had an intervention *vs* those who did not (Supplementary Table 2). We also conducted subgroup analysis of cases during the CDS period who had an intervention *vs* those who were identified by the model but did not receive an intervention (Supplementary Table 3).

### Statistical analyses

We summarised patient characteristics, clinical characteristics, and surgical variables across three time periods: pre-implementation, implementation, and post-implementation of the model. Categorical variables were reported as counts and percentages, while continuous variables were reported as mean (sd) or median (interquartile ranges; IQR) as appropriate based on distribution. Comparisons across time periods were conducted using χ^2^ tests for categorical variables and one-way analysis of variance (anova) for continuous variables. All statistical analyses were performed using R (version 4.4.3; R Foundation for Statistical Computing, Vienna, Austria), with a significance level α=0.05.

### Model performance

To assess discrimination of the implemented model compared with the 58-feature model and ASA physical status, we calculated the area under the receiver operating characteristic curve (AUROC) and area under the curve precision-recall (AUCPR). Note, ASA physical status was included as a comparator in order to provide a familiar reference point for contextualising model performance, not to suggest in any way (1) that mortality prediction is the intended purpose of the ASA physical status, or (2) that the aim of the present study was to compare model feasibility and clinical utility with that of ASA physical status.

Given the low prevalence of in-hospital mortality (1.56%), AUCPR was emphasised as a more informative metric in the setting of rare binary outcomes. ROC curve 95% confidence intervals (CIs) were computed using nonparametric bootstrap resampling via the DeLong method. For precision-recall curves, 95% CIs were derived from 100 bootstrap samples, with empirical percentiles computed at each recall threshold to construct shaded confidence bands. To further assess model performance, we computed classification metrics using the threshold that maximised Youden’s J statistic. These included accuracy, F1 score, precision (positive predictive value), recall (sensitivity), specificity, and the Brier score. The Brier score measures the mean squared difference between predicted probabilities and observed outcomes, with lower values indicating higher predictive accuracy. Model calibration was evaluated by dividing predicted probabilities into deciles and plotting observed *vs* predicted mortality rates. Comparative performance for both the implemented model and ASA physical status was assessed across the overall dataset, and within each implementation phase (pre-, during, and post-implementation). All analyses were conducted in R using the pROC, PRROC, yardstick, and ggplot2 packages.

### Time series analyses

To assess changes in model output and primary outcome rates over time, and whether the CDS tool was associated with changes in in-hospital mortality, we conducted exploratory segmented (interrupted) time series analyses. In order to obtain data for the time series analyses during the time periods before implementation, we applied the 32-feature model retrospectively to inpatients at RRUMC from February 2013 through April 2021. These data allowed us to accurately ascertain what the model output would have been for this 32-feature model before implementation. However all time series analyses were conducted as exploratory and descriptive, with the significant limitation regarding imputation of pre-implementation values, which limits the ability to draw causal or definitive inferences.

For the purposes of intervention time series analyses, the periods were divided into three phases: pre-CDS, CDS, and post-CDS. Monthly in-hospital mortality rates were analysed across these periods. We focused on patients identified as high risk by the prediction model (i.e. model output >0.5), representing the high-risk subgroup most likely to be impacted by the CDS. A secondary exploratory analysis included all surgical inpatients at RRUMC. For each outcome, we fit a segmented logistic regression model with terms representing time (continuous, in months), level shifts (binary indicators for the start of the CDS and post-CDS periods), and slope changes (terms between each post-intervention indicator). This approach allowed estimation of the baseline trend in mortality before CDS implementation, immediate changes in mortality level at each intervention point, and changes in trend (slope) after each intervention. Predicted mortality rates were extracted from the fitted model and overlaid against observed mortality rates for visualisation. Differences in trends or immediate effects between time segments were assessed using *P*-values for the relevant regression terms.

## Results

### Patient characteristics

**Fig 1.**
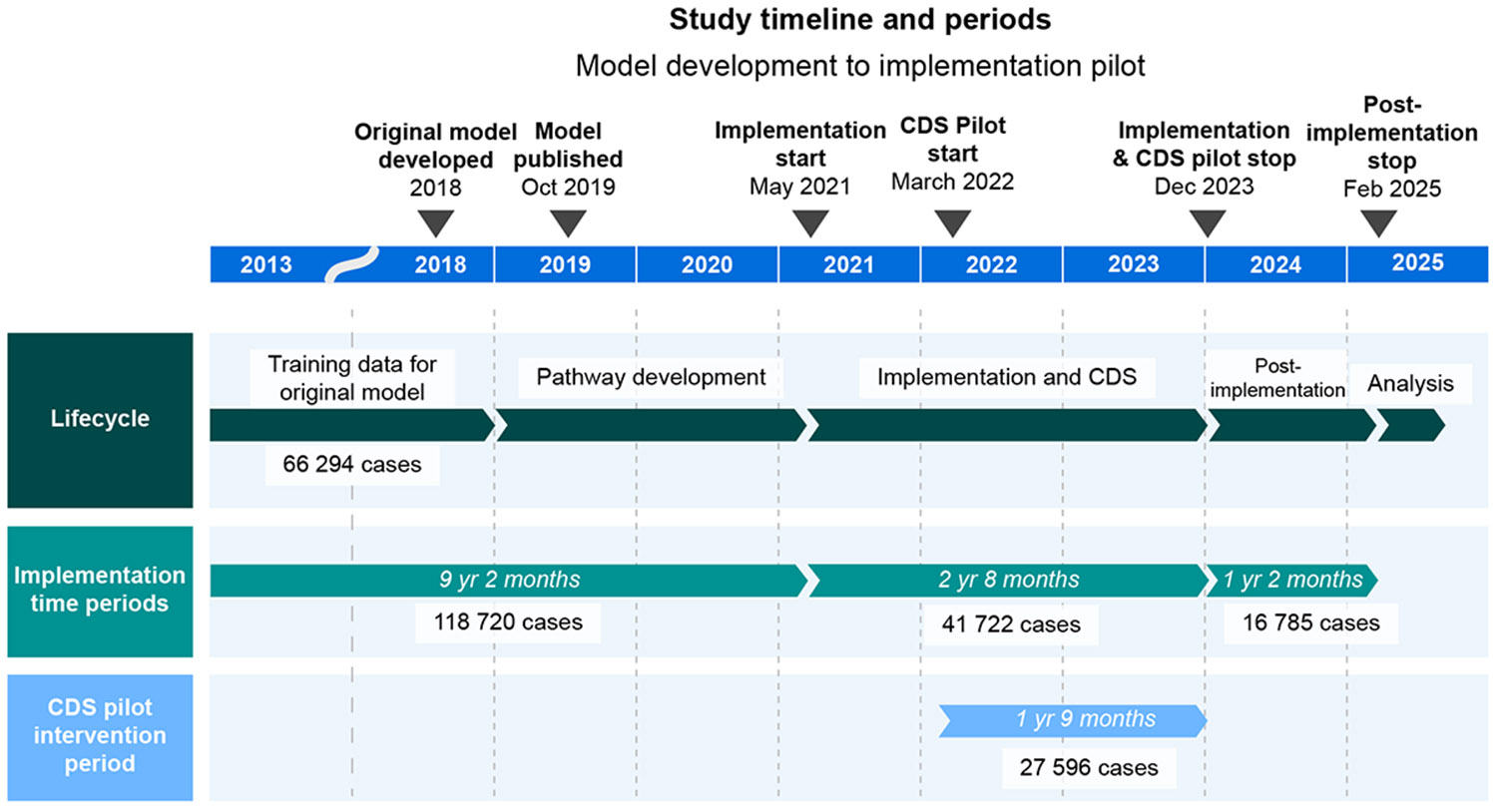
Study timeline and periods. CDS, clinical decision support.

From February 2013 to February 2025, the dataset included 177 227 cases, 175 491 unique encounters, and 134 115 patients. During the implementation period, there were 41 722 cases involving 25 018 patients. Study timeline and case numbers for each period are visualized in Figure 1. Overall mortality from 2013 to 2025 was 1.56%. The average patient age was 57 yr, and 48.4% were male. ASA physical status 3 was most common (49.8%), followed by ASA physical status 4 (11.9%). Racial/ethnic distribution included 20.1% Hispanic, 59% white, 9.9% Asian, and 7.4% black. Most cases (85.8%) were under general anaesthesia; 10.5% used sedation and 3.8% involved regional/neuraxial techniques. Postoperative acute kidney injury (AKI) occurred in 10.4%, median length of stay was 3 days, and 30-day readmission was 6.7%. Full details are in Table 1 and Figure 1.

### Implemented model performance

**Fig 2.**
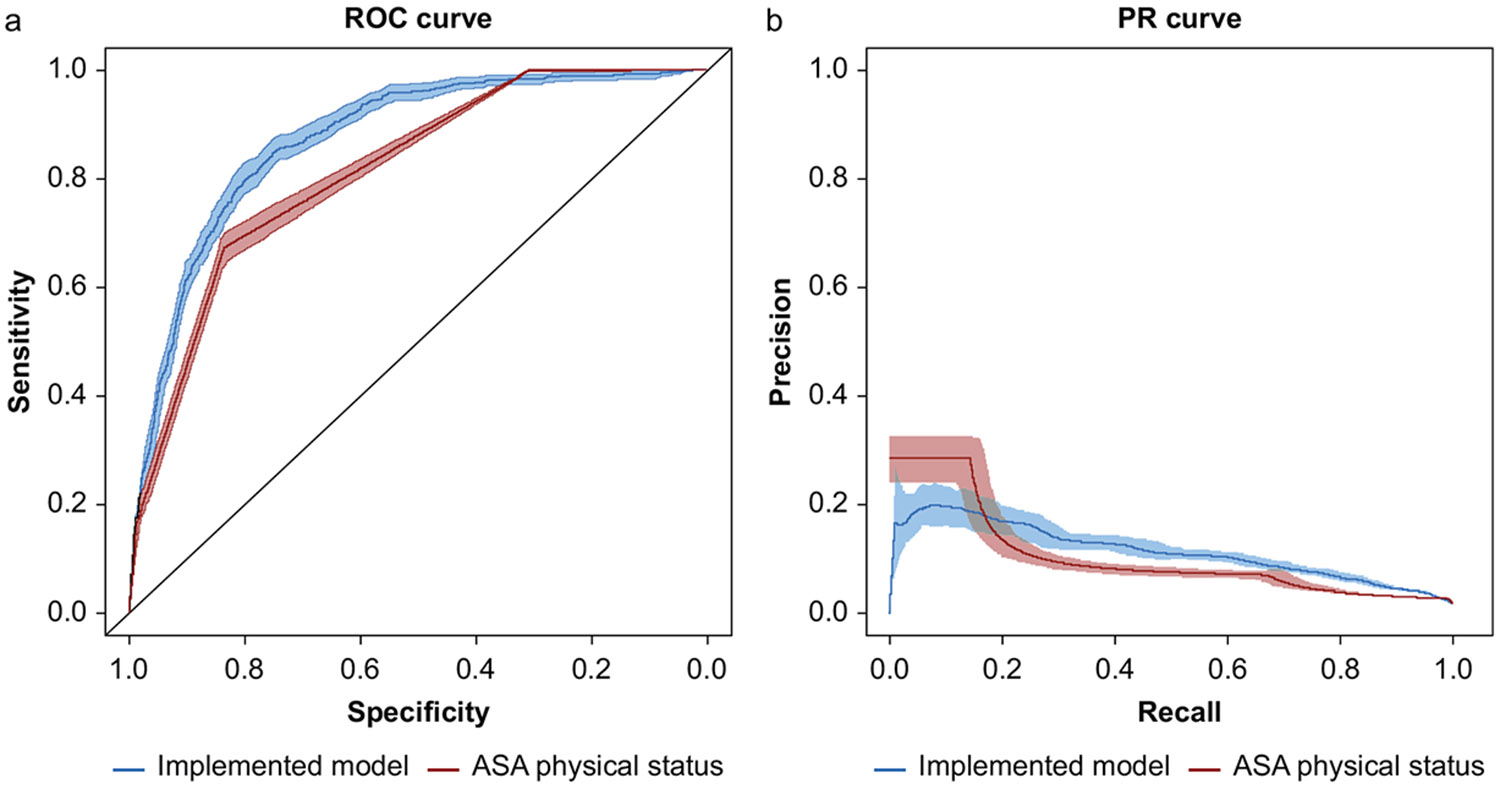
Performance of the Implemented Machine Learning Model compared with ASA physical status. (a) AUROC and (b) AUCPR of implemented model and ASA physical status. AUCPR, area under the curve precision-recall; AUROC, area under the receiver operating curve.

The implemented 32-feature model demonstrated strong performance with an AUROC of 0.870 (95% CI 0.858—0.881) and an AUCPR of 0.111 (Fig. 2, Table 2). The implemented model achieved an accuracy of 75.2%, an F1 score of 0.108, a precision of 0.058, a recall of 0.85, a specificity of 0.75, and a Brier score of 0.1242 (Fig. 2). In comparison, the ASA physical status-based model had a lower AUROC of 0.814 (95% CI 0.802—0.827) and AUCPR of 0.103, but a higher accuracy of 83.4%, an F1 score of 0.125, a precision of 0.069, a recall of 0.673, a specificity of 0.837, and a notably higher Brier score of 8.5449. Overall, the implemented model achieved better discrimination and calibration compared with the physician-assigned ASA physical status. The original 58-feature model, by comparison, demonstrated an AUC of 0.925 (95% CI 0.900—0.947), which was statistically significantly higher than the implemented model.

### Implementation feasibility

**Fig 3.**
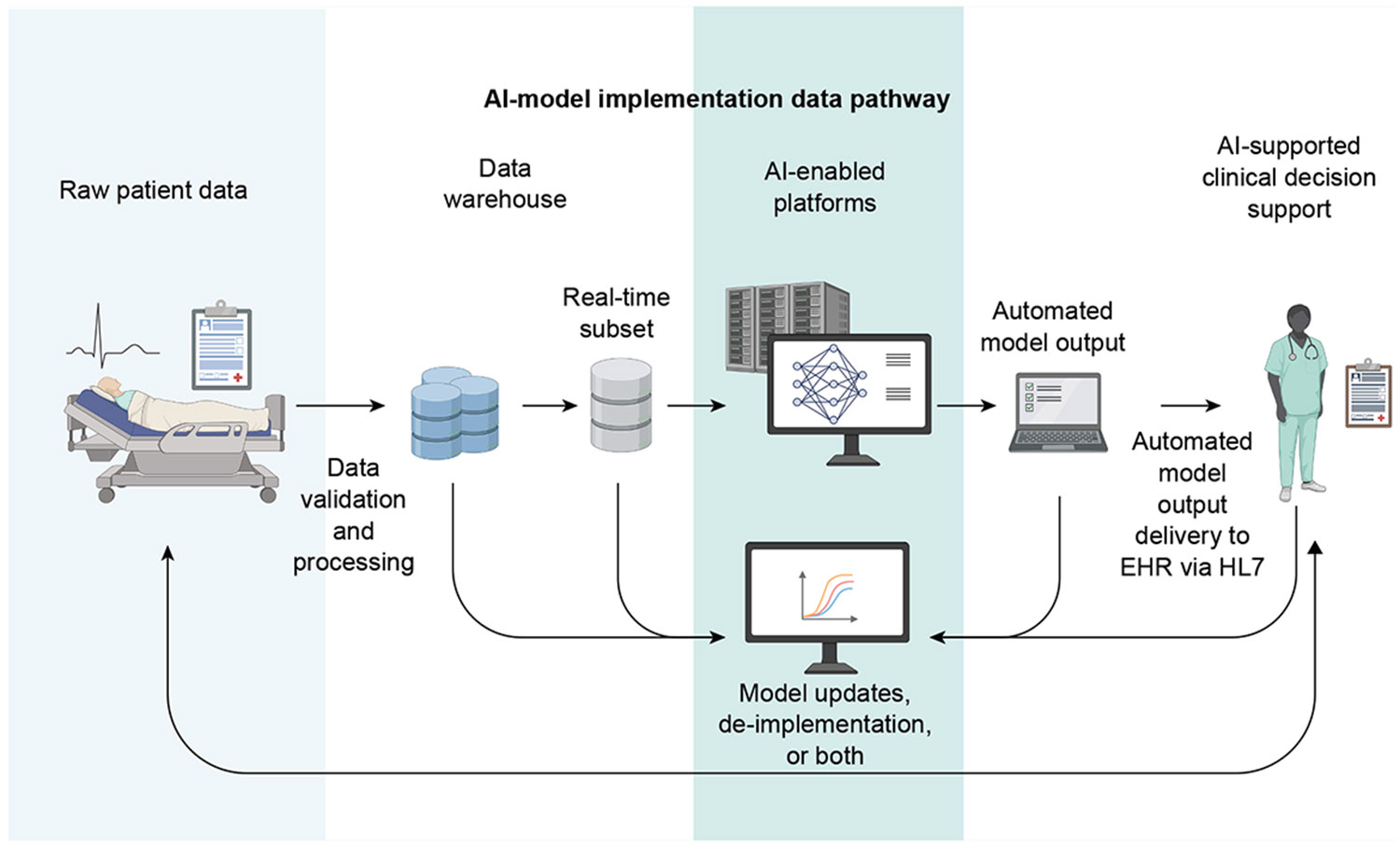
AI-model implementation data pathway. Created in BioRender. AI, artificial intelligence; EHR, electronic health record.

The implementation workflow is shown in Figure 3 and Supplementary Figure 1. Data were transferred for 3858 unique cases, with an average of 16.4 h between EHR data transfer and case start. The automated system successfully sent data for all eligible cases, including some that were later cancelled or deferred. Of the transferred cases, 2346 (60.8%) proceeded to surgery. Because the model output could change with updated inputs, many patients had multiple data transfers. Each case ID on average generated an average of 2.2 transfers. To give a sense of technical volume, we calculated an average of 9.5 model output transmissions to the EHR per day during the implementation period.

### Clinical decision support feasibility

During the intervention period, the voluntary survey had a 9.4% response rate. Respondents reported evaluating 260 cases, with interventions made in 35 (13.4%). Reported interventions included additional preoperative testing, additional preoperative optimisation, recommendation of a specific anaesthetic technique, and coordination of care with surgical services.

### Time series analyses

**Fig 4.**
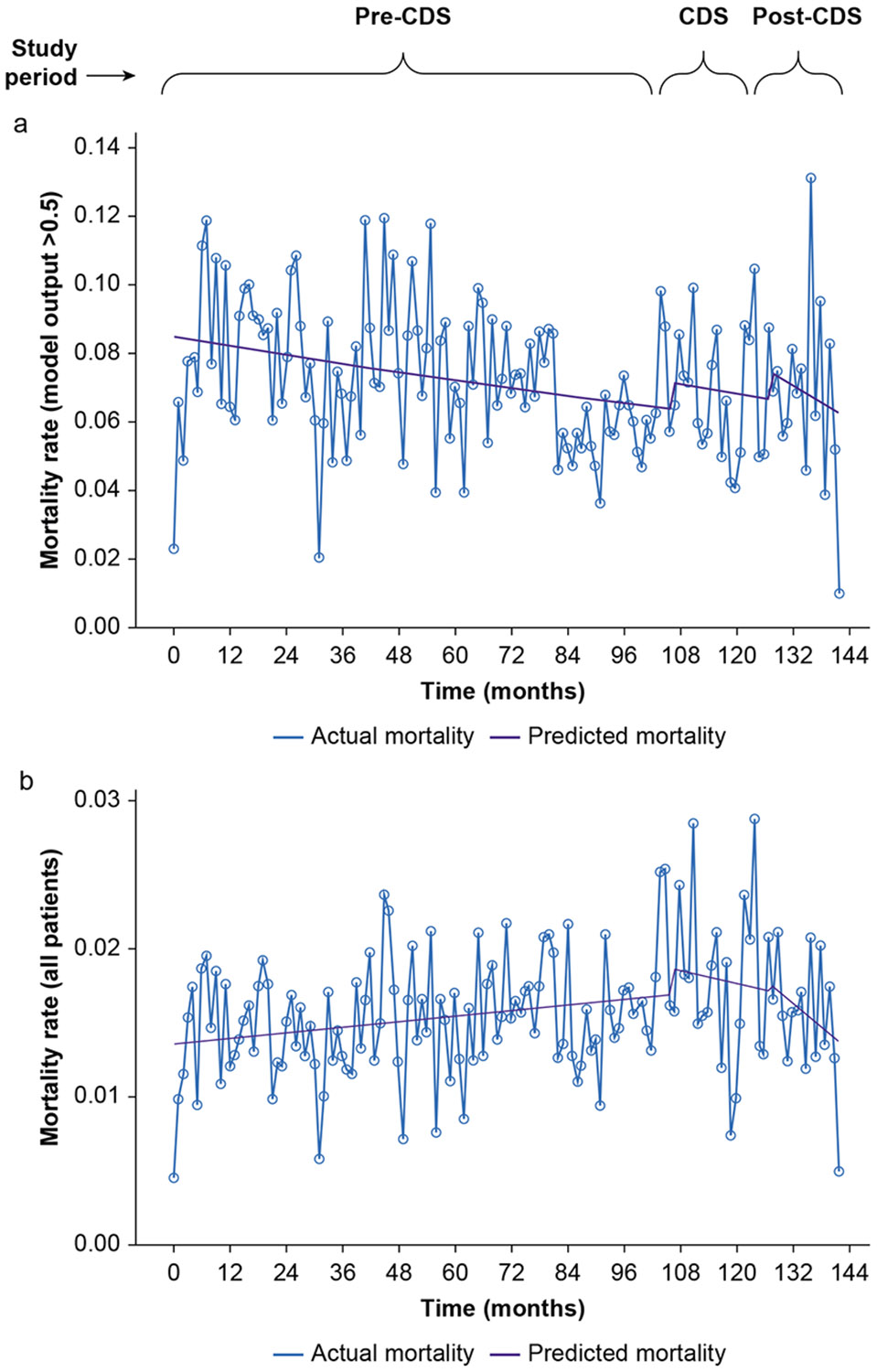
Time series of mortality among patients with mortality prediction model output >0.5 (a), and all patients (b). CDS, clinical decision sup.

We observed a significant downward trend in mortality among patients identified as high risk during the period before the CDS in our segmented regression time series analysis (odds ratio=0.997, 95% CI 0.995—0.999, *P*=0.001) (Fig. 4a). There were no significant changes in mortality during the CDS intervention period or after. No significant change in mortality rate was observed during or after CDS implementation.

In order to characterise baseline mortality over time, mortality was examined in all patients with segmented regression time series analysis (Fig. 4b). The period before the CDS showed a small but statistically significant increase in mortality risk over time (odds ratio=1.002, 95% CI 1.001—1.003, *P*=0.008). There were no significant changes in the slope or level of mortality during or after CDS implementation. We also examined the baseline proportion of cases identified as high risk by the model over time in order to understand changes over time in the patient population and model output. This showed an increase from 2013 to 2025 in patients identified as high risk (Supplementary Fig. 2). Establishing these baseline trends showed that mortality and high-risk status increased slightly over the time period studied.

### Exploratory clinical decision support tool cases with intervention

Subgroup analysis of cases during the CDS period who had an intervention or not demonstrated that patients with interventions tended to experience more postoperative complications (e.g. higher length of stay, AKI, transfer from ward to critical care) (Supplementary Table 2). We also conducted subgroup analyses of cases during the CDS period for patients who had an intervention or those who were identified by the model but did not receive an intervention (Supplementary Table 3), and similar findings were also seen. Cases with documented intervention were found to have worse outcomes and higher acuity, as evidenced by hospital length of stay, critical care length of stay, postoperative complications (e.g. AKI), and disposition. These subgroup analyses were exploratory in nature, and given the design limitations of the CDS tool, we caution against drawing any inferences from these results. Categories of interventions among patients who received interventions are summarized in Supplementary Table 4.

## Discussion

We present a prospective validation of a machine learning model for predicting postoperative mortality in surgical inpatients and demonstrate the feasibility of a pilot CDS tool. While a statistically significant reduction in AUROC was observed compared with the original model, the decline was modest and performance remained high. This study demonstrated feasibility of implementation of the ML model within the EHR, with reliable automated data transfers for all eligible cases and successful integration of model outputs into clinical workflows. The CDS pilot was used in a subset of cases, with documented clinician interventions based on model outputs, highlighting a pathway for potential utility in real-world practice.

This study highlights the importance of prospective validation and real-world implementation feasibility of ML or AI-based prediction models. Many researchers focus heavily on model development and retrospective performance; however few address the operational challenges of clinical integration. Our study demonstrates technical feasibility, high model performance, and potential for clinician engagement. It also addresses a critical gap in the literature between algorithm development and meaningful clinical impact.

As noted above, there were several differences between the original cohort used to train the model which significantly limits the study. The original model included all inpatients and outpatients undergoing surgery, whereas the implemented model only included inpatients at RRUMC. Implementing the model in this population was purposeful: (1) inpatients are known to have particularly high risk for postoperative mortality,^14^ (2) the CDS pilot was intended for the RRUMC location only to allow for controlled implementation with a small provider cohort, and (3), of the two UCLA medical centres, RRUMC is known to have a higher-acuity patient population. Additionally, this prospective evaluation only included one surgical encounter per patient, however, repeat surgeries were included in the original model dataset.^9^ Consequently, including repeat patients might have caused model performance to be slightly overestimated in the original model. Also, significantly fewer features (32 *vs* 58) were included in the implemented model, likely accounting for the observed reduction in performance.

Model drift is a risk for any study with prediction models spanning 10 years.^15,16^ Model drift is something that can occur over time as a result of changes in input variables, changes in the relationship between input variables and the outcome, changing clinical or documentation practices, and change in outcome prevalence.^17^ Time series analyses were undertaken in the present study, as these are important in identifying model drift and population drift. While most of the observed difference in performance between the implemented model and the original model was attributable to changes in the model and cohort, some effects could be related to temporal changes in the underlying outcome. Our time series analyses from 2013 to 2024 demonstrated a gradual increase in the primary outcome over time. In our case, most of the differences in performance observed are likely related to the differences in the original model and the implemented model, and not attributable to model drift.

Models that can trigger clinical interventions require careful consideration during recalibration.^18^ For example, if the CDS leads clinicians to change their practice for high-risk patients in a way that reduces deaths, this will lead to an apparent reduction in model performance. In this scenario, a recalibration would result in patients potentially no longer being identified as high risk, thereby preventing the clinicians from continuing to take the actions that were responsible for preventing deaths.

This study underlines several aspects important in machine learning operations (MLOps), an area which focuses on the deployment, monitoring, and maintenance of ML models in clinical settings.^5,19^ In particular, the reduction from 58 to 32 features was driven largely by technical and logistic feasibility, with regards to the availability and reliability of variables in real time before surgery. Unlike retrospective model-centric approaches, where data can be cleaned and validated manually at any pace, real-time implementation requires automated, high-speed systems for validation, processing, and data reduction. Preexisting high-quality, validated dataset, high-frequency data updates, and an AI-enabled platform were critical elements. MLOps promotes high-functioning implementation pathways such as this, where each layer is automated, fast, and with built-in quality control.^2,4^

Another important aspect highlighted by this study is how exactly AI and ML models are made available to providers. Effective implementation requires integration of human factors and implementation science, with emphasis on action-ability, safety, and utility.^1,6-8^ High-performing predictions alone are insufficient; models must fit in with existing work-flows and be associated with actionable, evidence-based clinical, technical, and administrative support.^20-22^ Clinician interventions in response to patients identified as high risk for mortality also exposes a significant challenge to mortality prediction models; if a patient is identified as high risk for inpatient mortality, often there is not a clear outcome-modifying action. Prediction models with outcomes associated with clearer clinical interventions are likely to provide more clinical value. Additionally, compliance and intervention rates were low in this study indicating low likelihood of clinical value during this pilot. While clinical value was not demonstrated here, one can argue that any interventions made highlights that there is potential for impact within ML prediction model implementation efforts. Prospective studies such as these demonstrate the need for future studies to identify barriers and pathways to effective use of AI-based CDS tools.^23^

This study has several limitations. While the AUROC of the original model was high, the AUCPR was comparatively low. In low-frequency outcomes, AUCPR might provide a more informative assessment of model performance, as it better reflects precision recall in imbalanced class distributions. Future model calibrations might be improved if AUCPR were optimised rather than AUROC. Additionally, the CDS tool was limited, purely passive, and served primarily to demonstrate technical feasibility rather than any evidence of clinical value. There were significant methodological limitations of this component of the study and future CDS efforts will require more robust design, active integration into clinical workflows, and outcome measures clearly able to evaluate clinical value. Future efforts must target maximisation of the potential impact of AI-based prediction models.^7,21,24,25^ Despite its limitations, this study represents an important step toward understanding real-world application of ML models in perioperative clinical practice.

We demonstrated feasibility of prospective validation of an automated ML model for predicting inpatient postoperative mortality, with acceptable performance metrics and EHR integration. Despite a modest decline from the original model performance, the implemented model maintained high AUROC and highlighted potential pathways for clinical utility. These results underscore the feasibility and importance of prospective validation, and draw attention to the work needed to reach the potential for ML models to inform clinical decision-making and improve perioperative care.

## Supplementary Material

Appendix and supplements

## Figures and Tables

**Table 1:** Patient characteristics. Time periods: pre-implementation, from February 2013 to April 2021; implementation, from May 2021 to December 2023; post-implementation, from June 2021 to February 2025 (see Fig. 1 for timelines). AKI, acute kidney injury; AMA, left against medical advice; MAC, monitored anaesthesia care; MI, myocardial infarction; SNF/REHAB, skilled nursing facility or rehabilitation centre.

	Time periods			*P*-value
	Pre-implementation	Implementation	Post-implementation	
Cases (*N*)	118 720	41 722	16 785	
Admissions (*N*)	118 709	40 018	16 764	
Patients (*N*)	93 879	25 018	15 218	
Mortality, *n* (%)	1769 (1.5)	740 (1.8)	261 (1.6)	<0.001
Age (yr)	56.9 (17.6)	57.6 (17.7)	58.1 (17.7)	<0.001
Male, *n* (%)	56 871 (47.9)	20 564 (49.3)	8304 (49.5)	<0.001
ASA physical status, *n* (%)				<0.001
1	6712 (5.7)	1233 (3.0)	395 (2.4)	
2	42 128 (35.5)	11 596 (27.8)	4561 (27.2)	
3	57 491 (48.4)	21 696 (52.0)	9097 (54.2)	
4	11 707 (9.9)	6821 (16.3)	2589 (15.4)	
5	682 (0.6)	376 (0.9)	143 (0.9)	
Implemented model output	0.2 (0.2)	0.3 (0.2)	0.3 (0.2)	<0.001
Race, *n* (%)				<0.001
White	72 861 (61.4)	22 661 (54.3)	9052 (53.9)	
Asian	11 390 (9.5)	4360 (10.5)	1793 (10.6)	
Black	8752 (7.4)	3161 (7.6)	1153 (6.9)	
Mideast	1629 (1.4)	1481 (3.5)	711 (4.2)	
Native	802 (0.7)	373 (0.9)	176 (1.0)	
Unknown	23 338 (19.7)	9686 (23.2)	3912 (23.3)	
Ethnicity, *n* (%)				<0.001
Hispanic	22 237 (18.7)	9502 (22.8)	3841 (22.9)	
Non-Hispanic	90 444 (76.2)	29 133 (69.8)	11 537 (68.7)	
Unknown	6039 (5.1)	3087 (7.4)	1407 (8.4)	
Postoperative AKI, *n* (%)	12 354 (10.4)	4478 (10.7)	1666 (9.9)	0.013
Postoperative MI, *n* (%)	20 (0.0)	10 (0.0)	6 (0.0)	0.196
Length of stay (median days)	3.0 (1.0, 6.0)	3.0 (1.0, 7.0)	3.0 (1.0, 7.0)	<0.001
ICU hours (mean)	40.7 (194.3)	48.3 (222.0)	37.7 (172.7)	<0.001
ICU hours (median)	0.0 (0.0, 0.0)	0.0 (0.0, 0.0)	0.0 (0.0, 0.0)	
Readmission 30 day, *n* (%)	8067 (6.8)	2877 (6.9)	1003 (5.9)	<0.001
Anaesthesia type, *n* (%)				<0.001
General	100 068 (84.5)	36 602 (88.4)	14 712 (88.2)	
MAC	12 889 (10.9)	3989 (9.6)	1623 (9.7)	
Regional/neuraxial	5509 (4.7)	799 (1.9)	350 (2.1)	
Case service, *n* (%)				<0.001
General surgery	20 125 (16.9)	7183 (17.2)	2881 (17.0)	
Orthopaedics	15 887 (13.3)	4895 (11.7)	1962 (11.6)	
Urology	15 095 (12.6)	4812 (11.5)	2018 (11.9)	
Gastroenterology	9489 (7.9)	4733 (11.3)	1741 (10.3)	
Neurosurgery	10 454 (8.8)	3839 (9.2)	1621 (9.6)	
Otolaryngology	9071 (7.6)	2870 (6.9)	1183 (7.0)	
Obstetrics and gynaecology	7559 (6.3)	2091 (5.0)	695 (4.1)	
Cardiac surgery	6460 (5.4)	2088 (5.0)	799 (4.7)	
Plastic surgery	3734 (3.1)	1716 (4.1)	814 (4.8)	
Cardiology	3739 (3.1)	1800 (4.3)	759 (4.5)	
Vascular surgery	3888 (3.3)	983 (2.4)	322 (1.9)	
Surgical oncology	2897 (2.4)	1112 (2.7)	534 (3.2)	
Thoracic surgery	2774 (2.3)	970 (2.3)	604 (3.6)	
Financial status				<0.001
Private	60 159 (50.5)	19 013 (45.6)	7594 (44.8)	
Medical	53 980 (45.3)	21258 (51.0)	8816 (52.0)	
Other	3396 (2.8)	985 (2.4)	360 (2.1)	
Tricare	895 (0.8)	225 (0.5)	96 (0.6)	
Workers comp	804 (0.7)	222 (0.5)	72 (0.4)	
Missing	144	24	13	
Disposition, *n* (%)				<0.001
Home	81 146 (68.0)	29 296 (70.2)	12 065 (71.3)	
Home health	22 838 (19.1)	6689 (16.0)	2795 (16.5)	
SNF/REHAB	11 130 (9.4)	3976 (9.5)	1456 (8.6)	
AMA	199 (0.2)	129 (0.3)	70 (0.4)	
Expired	1681 (1.4)	718 (1.7)	264 (1.6)	
Hospice	548 (0.5)	177 (0.4)	55 (0.3)	
Hospital	1050 (0.9)	459 (1.1)	160 (0.9)	
Law enforcement	13 (0.0)	4 (0.0)	4 (0.0)	
Longterm care	533 (0.4)	234 (0.6)	44 (0.3)	
Psychiatric unit or hospital	153 (0.1)	44 (0.1)	13 (0.1)	
Missing	87	1	25	

**Table 2:** In-hospital mortality machine learning model performance characteristics. AUCPR, area under the curve precision-recall; AUROC, area under the receiver operating curve. AUROC shown with confidence intervals. Performance metrics calculated based on the threshold that maximised the Youden’s J statistic for each model.

	AUROC	AUCPR	Accuracy	F1	Precision	Recall	Specificity	Brier
Implemented model	0.870 (0.858—0.881)	0.111	0.752	0.108	0.058	0.85	0.75	0.1242
ASA physical status	0.814 (0.802—0.827)	0.103	0.834	0.125	0.069	0.673	0.837	8.5449

## Editor’s key points

- Machine learning prediction models for use in perioperative clinical decision-making require prospective validation to ensure implementation fidelity and feasibility.
- The authors prospectively validated a postoperative mortality prediction model in inpatients undergoing surgery at a US academic medical centre and evaluated feasibility for clinical decision support.
- Prospective validation and feasibility were shown for an automated machine learning model for predicting inpatient postoperative mortality as a step towards establishing machine learning models for informing clinical decision-making in perioperative care.

## Appendix A. Supplementary data

Supplementary data to this article can be found online at https://doi.org/10.1016/j.bja.2025.11.042.
