# Supplementary material for: Prospective validation and real-time implementation of an automated machine learning postoperative mortality prediction model
Source: Br J Anaesth. Author manuscript; Available in PMC 2026 Feb 24. (PMC12927408; doi:10.1016/j.bja.2025.11.042)
Supplement: Appendix and supplements [file NIHMS2141045-supplement-Appendix_and_supplements.pdf]

SUPPLEMENTARY MATERIALS

Supplementary Table 1: Implemented Model Feature List

Feature	Percentage of Data Missing	Data Type
Age	0	Continuous
Albumin	34.7	Continuous
Alkaline Phosphatase	29.0	Continuous
ALT	27.9	Continuous
AST	27.3	Continuous
Bicarbonate	88.5	Continuous
Bilirubin Total	31.9	Continuous
Body Mass Index	2.0	Continuous
BNP	84.7	Continuous
Case Service Name	1.1	Categorical (46)
Chloride	11.5	Continuous
Creatinine	19.7	Continuous
Diastolic Blood Pressure	0.3	Continuous
Diastolic Pulmonary Artery Pressure	97.5	Continuous
Echo Ejection Fraction	73.7	Continuous
Gender	0.0	Categorical (4)
Glucose	19.0	Continuous
Height (inches)	2.0	Continuous
Hemoglobin	19.7	Continuous
INR	27.6	Continuous
Patient Class	0.0	Categorical (6)
Platelet Count	21.3	Continuous
Potassium	57.9	Continuous
Prothrombin Time	27.9	Continuous
Pulse	0.3	Continuous
Sodium	11.4	Continuous
SpO2	0.6	Continuous
Systolic Blood Pressure	0.3	Continuous
Systolic Pulmonary Artery Pressure	97.5	Continuous
Urea Nitrogen	12.4	Continuous

Weight (kilograms)	0.0	Continuous
White Blood Cell Count	22.0	Continuous

Preoperative features used in the model and percent missingness. Feature values were either continuous or categorical; the number of possible variables for each categorical variable is shown in parenthesis. For physiologic features the most recent value prior to surgery was obtained. Features used in the original model can be found in detail.[8]

Supplementary Figure 1. Database Structure and Pathways

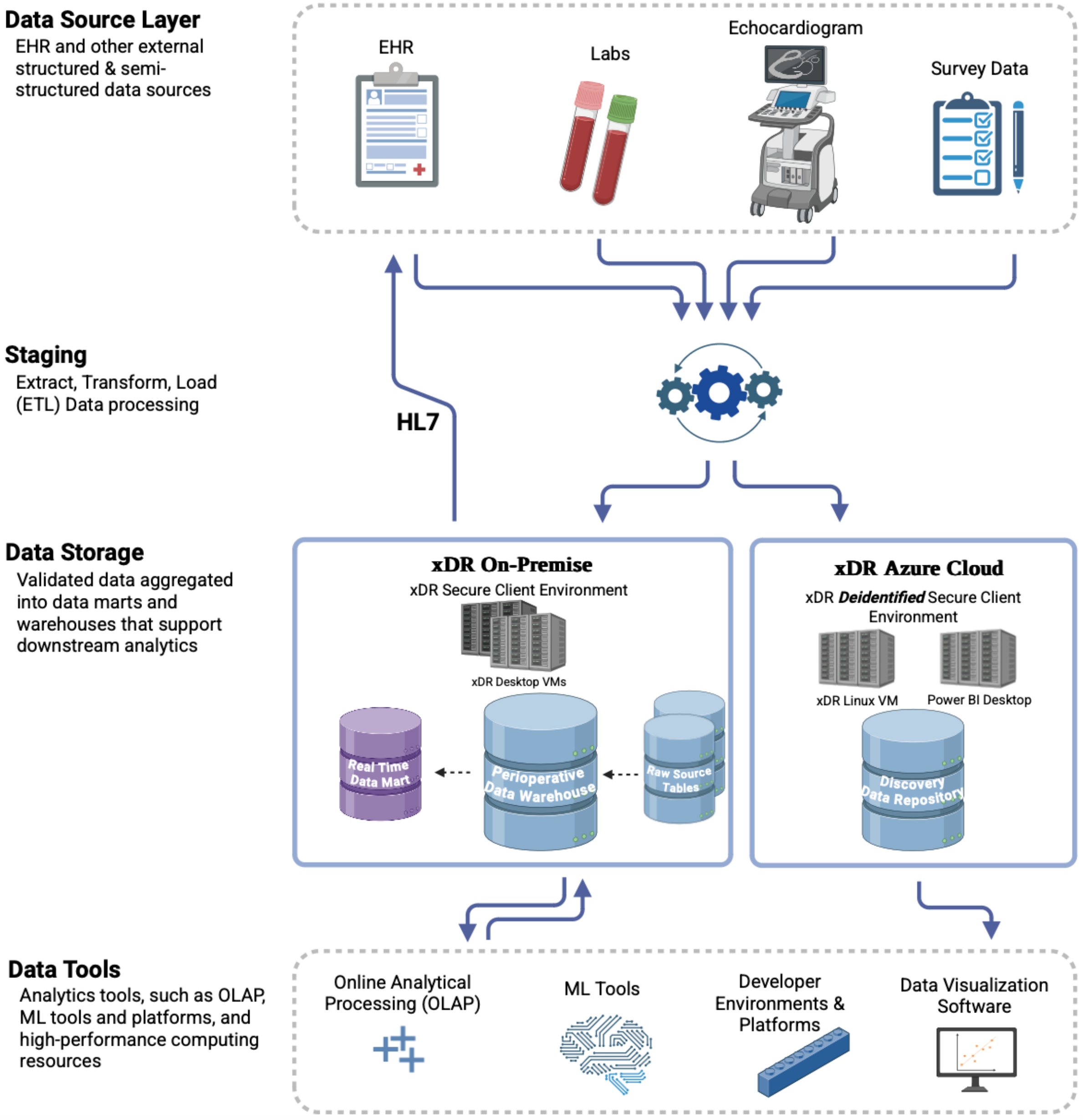

Figure Legend: A diagram of the institutional data architecture and integration pathways is provided in the above Supplementary Figure 1. Figure Created with BioRender.

Clinical source data from electronic health record, echocardiogram databases, and other external sources are organized via ETL processes into validated tables within the Data Storage Layer. The data storage layer includes both identified on-premise databases and deidentified cloud-based databases. Implementation processes all work through the identified data storage elements (xDR On-Premise). The deidentified databases are ideal for exploratory work and were used for the initial model development. The On-Premise environment takes data from electronic health record source tables and places them in data warehouses (such as the Perioperative Data Warehouse)

and the data marts (such as the Real Time Data Mart). The Real Time data mart received data feeds at 6-hour intervals using Fast Healthcare Interoperability Resources (FHIR) and represents a smaller use-specific set of variables. This real-time data mart integrates multiple upstream sources, including ADT (admission, discharge, transfer) feeds, laboratory systems, and structured clinical data.

These pipelines feed into the model inference engine in the Data Tools layer, which re-executes the model whenever updated data are available. Model inference was executed automatically upon data receipt using Python within a Jupyter Notebook environment (v6.4.5), running on a virtual machine provisioned through institutional IT infrastructure. The virtual machine (VM) is coordinated by the UCLA Office of Health Informatics and Analytics. Model output calculation was computationally efficient and did not rely on GPU acceleration or high-performance computing resources, underscoring the feasibility of running the model within standard clinical computing environments.

In order for this particular model to run, some variables came directly from minimally processed Clarity tables (i.e. Patient ID, case ID, scheduled surgery date) and the other model input variables came from the Real Time Data Mart. Numerous tools are available to end users, including online analytic processing (e.g. Tableau), ML tools (e.g. Python, R, Jupyter), Developer Environments and platforms (e.g. Power BI, Databricks), Data visualization tools, among other tools enabled with high-performance computing resources.

Model outputs were transmitted at regular intervals back to the EHR via an HL7 interface as a dichotomous variable, automatically defaulting to null when no high-risk designation was present.

Supplementary Figure 2. Proportion of patients identified as High-risk over Time

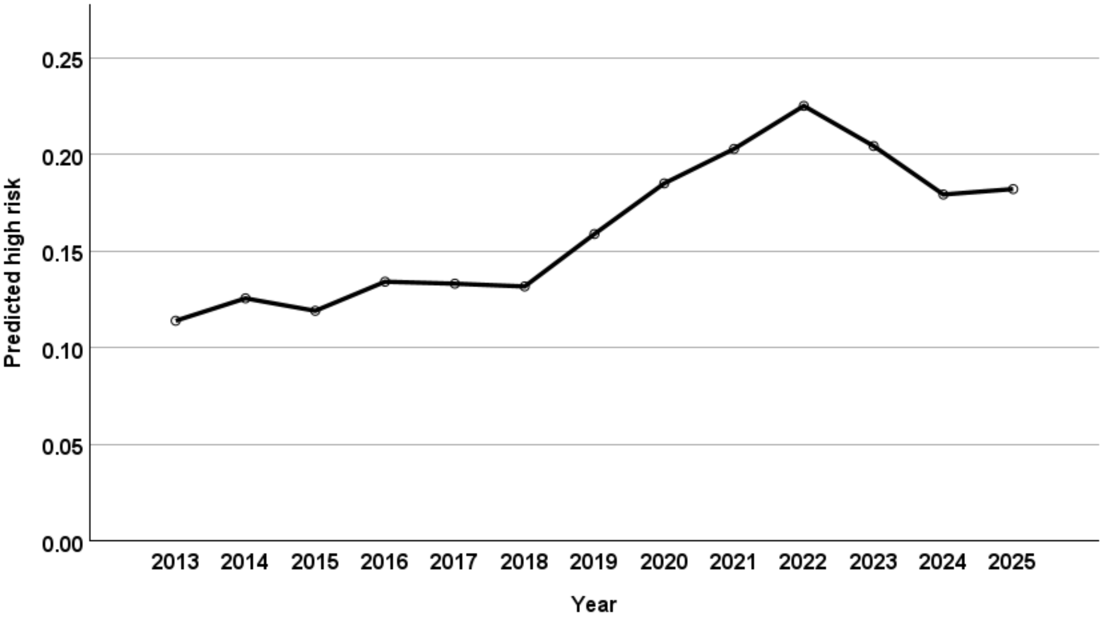

Supplementary Table 2. Patient Characteristics of Cases with CDS Intervention vs All Other Case During CDS Time Period (March 2022-Dec 2023)

	**CDS Period**		
	**No**	**Yes**	
**Cases (N)**	**27,580**	**N=14**	**P-value**
**Age**	57.6 (17.7)	57.5 (16.3)	0.981
**Male**	13609 (49.3%)	6 (42.9%)	0.79
**ASA Status**			
1	746 (2.7%)	0	0.007
2	7453 (27.0%)	1 (7.1%)	
3	14313 (51.9%)	5 (35.7%)	
4	4803 (17.4%)	7 (50.0%)	
5	265 (1.0%)	1 (7.1%)	
**Implemented Model Output**	0.3 (0.2)	0.6 (0.2)	<0.001
**Mortality**	492 (1.8%)	0	1
**Postoperative AKI (Any)**	3019 (10.9%)	6 (42.9%)	0.002
**Postoperative AKI Stage**			
1	1822 (6.6%)	1 (7.1%)	<0.001
2	303 (1.1%)	2 (14.3%)	
3	894 (3.2%)	3 (21.4%)	
**Postoperative MI**	7 (0.0%)	0	1
**Length of Stay (mean days)**	7.5 (15.5)	55.0 (57.2)	<0.001
**Length of Stay (median days)**	3.0 (1.0, 7.0)	37.5 (8.0, 95.2)	<0.001
**ICU Hours (Mean)**	48.9 (227.8)	865.4 (1072.7)	<0.001
**ICU Hours (Median)**	0.0 (0.0, 0.0)	272.3 (0.0, 1411.7)	<0.001
**Readmission 30 day**	1877 (6.8%)	0	0.619
**Postoperative Floor to ICU transfer**	592 (2.1%)	5 (35.7%)	<0.001
**Disposition**			
HOME	19317 (70.0%)	4 (28.6%)	<0.001
HOME HEALTH	4532 (16.4%)	2 (14.3%)	
SNF/REHAB	2564 (9.3%)	4 (28.6%)	
AMA	94 (0.3%)	0	
EXPIRED	474 (1.7%)	0	
HOSPICE	115 (0.4%)	1 (7.1%)	
HOSPITAL	286 (1.0%)	0	
LAW ENFORCEMENT	1 (0.0%)	0	
LONGTERM CARE	168 (0.6%)	3 (21.4%)	
PSYCH	28 (0.1%)	0	

**Race**			
White	14888 (54.0%)	9 (64.3%)	
Asian	2950 (10.7%)	2 (14.3%)	
Black	2079 (7.5%)	0	0.639
Mideast	1065 (3.9%)	1 (7.1%)	
Native	251 (0.9%)	0	
Unknown	6347 (23.0%)	2 (14.3%)	
**Ethnicity**			
Hispanic	6432 (23.3%)	2 (14.3%)	0.657
Non-Hispanic	19179 (69.5%)	11 (78.6%)	
Unknown	1969 (7.1%)	1 (7.1%)	
**Anesthesia Type**			
General	24167 (88.3%)	11 (78.6%)	0.145
MAC	2646 (9.7%)	2 (14.3%)	
Regional/Neuraxial	550 (2.0%)	1 (7.1%)	
**Case Service**			
General Surgery	4791 (17.4%)	1 (7.1%)	
Orthopaedics	3179 (11.5%)	1 (7.1%)	
Urology	3212 (11.6%)	1 (7.1%)	
Gastroenterology	3145 (11.4%)	1 (7.1%)	
Neurosurgery	2608 (9.5%)	2 (14.3%)	
Otolaryngology	1889 (6.8%)	1 (7.1%)	
Obstetrics and Gynecology	1256 (4.6%)	0	0.005
Cardiac Surgery	1425 (5.2%)	1 (7.1%)	
Plastic Surgery	1142 (4.1%)	1 (7.1%)	
Cardiology	1173 (4.3%)	2 (14.3%)	
Vascular Surgery	647 (2.3%)	1 (7.1%)	
Surgical Oncology	727 (2.6%)	0	
Thoracic Surgery	662 (2.4%)	0	

Abbreviations: Monitored Anesthesia Care (MAC), Myocardial infarction (MI), Acute Kidney Injury (AKI), Left Against Medical Advice (AMA), Skilled Nursing Facility or Rehabilitation center (SNF/REHAB), psychiatric unit or hospital (PSYCH). See Figure 1 for timeframes.

Supplementary Table 3. Patient Characteristics of Cases with CDS Intervention vs Cases Identified as "High-Risk" During CDS Time Period (March 2022-Dec 2023)

	CDS Period, High-Risk Cases Only		
	No	Yes	
**Cases (N)**	**5,825**	**N=12**	**P-value**
**Age**	60.3 (17.5)	59.0 (17.0)	0.795
**Male**	3393 (58.2%)	6 (50.0%)	0.571
**ASA Status**			
1	14 (0.2%)	0	0.331
2	209 (3.6%)	0	
3	2802 (48.1%)	4 (33.3%)	
4	2650 (45.5%)	7 (58.3%)	
5	150 (2.6%)	1 (8.3%)	
**Implemented Model Output**	0.7 (0.1)	0.7 (0.1)	0.742
**Mortality**	399 (6.8%)	0	1
**Postoperative AKI (Any)**	1909 (32.8%)	6 (50.0%)	0.225
**Postoperative AKI Stage**			
1	1016 (17.4%)	1 (8.3%)	0.046
2	231 (4.0%)	2 (16.7%)	
3	662 (11.4%)	3 (25.0%)	
**Postoperative MI**	0	0	1
**Length of Stay (mean days)**	20.5 (27.4)	63.8 (57.2)	<0.002
**Length of Stay (median days)**	12.0 (6.0, 24.0)	56.5 (17.0, 108.8)	<0.002
**ICU Hours (Mean)**	177.1 (452.1)	1009.7 (1095.9)	<0.001
**ICU Hours (Median)**	0.0 (0.0, 144.9)	638.1 (104.6, 1628.6)	<0.001
**Readmission 30 day**	867 (14.9%)	0	0.234
**Postoperative Floor to ICU transfer**	416 (7.1%)	5 (41.7%)	<0.001
**Disposition**			
HOME	2230 (38.3%)	2 (16.7%)	0.007
HOME HEALTH	1659 (28.5%)	2 (16.7%)	
SNF/REHAB	1116 (19.2%)	4 (33.3%)	
AMA	43 (0.7%)	0	
EXPIRED	384 (6.6%)	0	
HOSPICE	86 (1.5%)	1 (8.3%)	
HOSPITAL	165 (2.8%)	0	
LAW ENFORCEMENT	1 (0.0%)	0	
LONGTERM CARE	132 (2.3%)	3 (25.0%)	

PSYCH	9 (0.2%)	0	
**Race**			
White	2650 (45.5%)	8 (66.7%)	0.235
Asian	587 (10.1%)	2 (16.7%)	
Black	622 (10.7%)	0	
Mideast	235 (4.0%)	1 (8.3%)	
Native	53 (0.9%)	0	
Unknown	1678 (28.8%)	1 (8.3%)	
**Ethnicity**			
Hispanic	1890 (32.4%)	1 (8.3%)	0.118
Non-Hispanic	3657 (62.8%)	10 (83.3%)	
Unknown	278 (4.8%)	1 (8.3%)	
**Anesthesia Type**			
General	4319 (75.0%)	11 (91.7%)	0.37
MAC	1399 (24.3%)	1 (8.3%)	
Regional/Neuraxial	39 (0.7%)	0	
**Case Service**			
General Surgery	410 (7.0%)	1 (8.3%)	0.014
Orthopaedics	305 (5.2%)	1 (8.3%)	
Urology	208 (3.6%)	1 (8.3%)	
Gastroenterology	2134 (36.6%)	1 (8.3%)	
Neurosurgery	365 (6.3%)	2 (16.7%)	
Otolaryngology	151 (2.6%)	1 (8.3%)	
Obstetrics and Gynecology	47 (0.8%)	0	
Cardiac Surgery	493 (8.5%)	1 (8.3%)	
Plastic Surgery	16 (0.3%)	0	
Cardiology	512 (8.8%)	2 (16.7%)	
Vascular Surgery	163 (2.8%)	1 (8.3%)	
Surgical Oncology	82 (1.4%)	0	
Thoracic Surgery	58 (1.0%)	0	

Abbreviations: Monitored Anesthesia Care (MAC), Myocardial infarction (MI), Acute Kidney Injury (AKI), Left Against Medical Advice (AMA), Skilled Nursing Facility or Rehabilitation center (SNF/REHAB), psychiatric unit or hospital (PSYCH). See Figure 1 for timeframes.

Supplementary Table 4 Provider Responses to the Survey question: “Select any/all interventions taken for the patient”

Intervention Category	Number
Additional preoperative testing (cardiac workup, CT scan, etc.)	2
Additional preoperative optimization (initiation/continuation of beta blocker, tapering pain medication, etc)	2
Additional nonstandard intraoperative monitors recommended (eg PA catheter, TEE)	0
Recommendation of specific anesthetic technique (eg local, neuraxial, regional)	1
Assignment of specific provider/team (eg cardiac anesthesia, senior resident)	0
Recommendation of timing of procedure (eg first case start)	2
Postoperative monitoring (disposition, EtCO2, etc.)	0
Other	4
Intervention not entered	24
Total	35

Supplementary Materials 1. Survey Instrument: Questions and Format

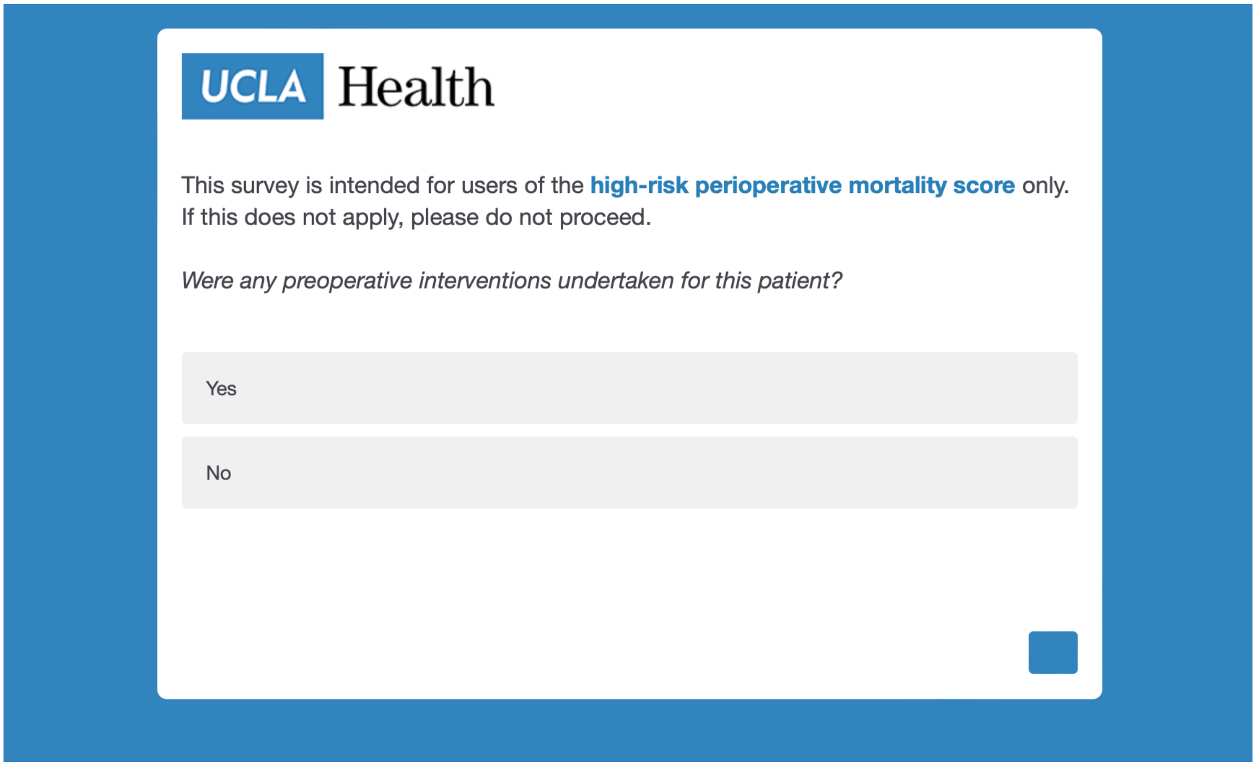

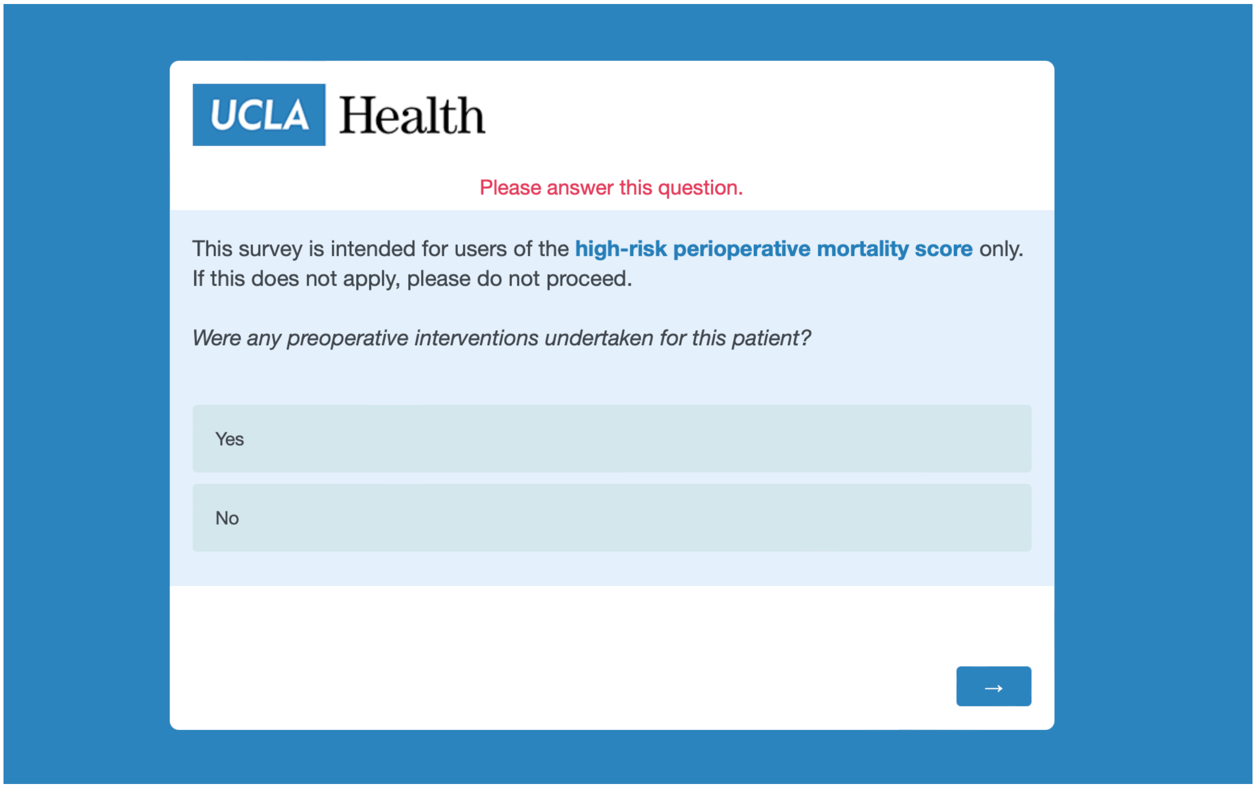

UCLA Health

Select any/all interventions taken for the patient:

- Additional preoperative testing (cardiac workup, CT scan, etc.)
- Additional preoperative optimization (initiation/continuation of beta blocker, tapering pain medication, etc)
- Additional nonstandard intraoperative monitors recommended (eg PA catheter, TEE)
- Recommendation of specific anesthetic technique (eg local, neuraxial, regional)
- Assignment of specific provider/team (eg cardiac anesthesia, senior resident)
- Recommendation of timing of procedure (eg first case start)
- Postoperative monitoring (disposition, EtCO2, etc.)
- Other

→

Supplementary Materials 2. Python code for Random Forest Mortality Prediction Model.

```
#!/usr/bin/env python
# coding: utf-8

# In[1]:


import os
import sys
import re
from tempfile import mkdtemp

print(sys.version_info)

home_dir = os.getenv("HOME")
print(os.getenv("PYTHONPATH"))

import numpy as np
import pickle
import pandas as pd
import matplotlib.pyplot as plt
import scipy.stats as st

home_dir = os.getenv("HOME")
print(os.getenv("PYTHONPATH"))

import numpy as np
import pickle
import pandas as pd
import matplotlib.pyplot as plt
import scipy.stats as st
import hashlib
import pyodbc

from sklearn import metrics, tree
from sklearn.impute import SimpleImputer
from sklearn.preprocessing import StandardScaler, LabelEncoder
from sklearn.metrics import precision_recall_curve, average_precision_score, brier_score_loss,
make_scorer
from sklearn.linear_model import LogisticRegression, ElasticNet, ElasticNetCV,
ARDRegression, SGDClassifier
from sklearn.ensemble import RandomForestClassifier
from sklearn.base import BaseEstimator, TransformerMixin
import joblib
from datetime import datetime
```

```
#from fancyimpute import SoftImpute
from ehr_utils import *
#from xgboost import XGBClassifier

# # Configurations
# - main_filtered_f - this is the "main" data file, which we assume contains all features needed
for prediction
#
# - test_or_case_id_f - this file contains all the OR_CASE_IDs that we want to predict the
mortality risk for
#
# - exp_prefix - this corresponds to one of the directories in "paper" and this determines which
set of features to use to predict mortality.
#
# - dir_to_save_files - this should usually be the full path to the directory that contains
EHR_MAIN_FEATURES.csv and this is where all output results will be saved
#
# - data_dir - this directory contains a file per each categorical variable, and each file contains the
allowable values for each variable
#
# - model_dir - this is the directory that contains the binary ML models

# In[2]:


# set to False to hide IDs
verbose = True

# version of scikit-learn that was used to create model (should be in pickled model filename)
sk_version = "0.21.3"

## this variable is the column that we will use as the target variable for the model
TARGET_VARIABLE = 'INPT_DEATH_YN'

MIN_ASA_STATUS=1
MAX_ASA_STATUS=5
MIN_AGE=18
MAX_AGE=89

# PATH SETTINGS
#main_filtered_f = "main_Nov21_2017_Feb_13_2018.filtered.main.txt"
main_filtered_f = "/opt/data/workingdir/blhill/main_merged_w_akin_spo2.filtered.main.txt"
#main_filtered_f = "/opt/genomics/workingdir/blhill/test_main_3.txt"
test_or_case_id_f = "/opt/data/workingdir/blhill/or_case_id_032018.txt"
#main_filtered_f = "/opt/genomics/workingdir/blhill/vali_test.txt"
```

```
# directory containing code repo
repo_dir = "\\\SSPFPGDS04\ValiPython\TestPythonProg"

# experiment prefix sets the set of features to use in the model
exp_prefix = "preop_no_lab_times"
dir_to_save_files = os.path.join(repo_dir, "paper", exp_prefix)

# directory coaining info about acceptable data
data_dir = os.path.join(repo_dir, "data")

## set path to directory containing pickled models
#model_dir = os.path.join("/opt/genomics/workingdir/blhill/mortality_models", exp_prefix)
model_dir = dir_to_save_files

if not os.path.exists(dir_to_save_files):
    os.makedirs(dir_to_save_files)


# In[3]:

connection = pyodbc.connect("Driver={ODBC Driver 13 for SQL Server};"
                      "Server=uclaedrprd;"
                      "Database=PerioperativeDM;"
                      "Trusted_Connection=yes;")
cursor = connection.cursor()
#cursor.execute("INSERT INTO PerioperativeDM.SCRATCH.Mortality_Prediction VALUES
(?,?,?,?,?,?)", 1, 1, 1, 1,datetime.today(),'1')
#onnection.commit()

print(connection)

if connection:
    print("Yes, we are connected \n")


sql = '''SELECT distinct * FROM PerioperativeDM.SCRATCH.Mortality_inp3
'''

#Importing from SQL into Dataframe
df= pd.read_sql(sql,connection)
print(df.head())


# # Read in data to data frame
```

```
# In[4]:


#df = pd.read_csv(os.path.join(home_dir, main_filtered_f), sep="|", header=0)
print(df.shape)
print(len(df.columns))
#if verbose:
#    display(df.iloc[0:20, :])



# make sure INPT_DEATH_YN is set to boolean
df[TARGET_VARIABLE] = df[TARGET_VARIABLE].astype(bool)


# # hash OR_CASE_ID values and take only patients after March 2018

# In[ ]:


#cursor.execute("INSERT INTO PerioperativeDM.SCRATCH.Mortality_Prediction VALUES
(?,?,?,?,?,?)", 1, 1, 1, 1,datetime.today(),'1.6')
#connection.commit()


# In[5]:


#def get_sha256_hash(x):
#    m = hashlib.sha256()
#    m.update(str(x).encode('utf-8'))
#    return m.hexdigest().upper()

#df["OR_CASE_ID"] = df["OR_CASE_ID"].apply(get_sha256_hash)


#df = df[df["OR_CASE_ID"].isin(test_or_case_ids.iloc[:,0])]


# # drop any rows that are exact copies of another row

#cursor.execute("INSERT INTO PerioperativeDM.SCRATCH.Mortality_Prediction VALUES
(?,?,?,?,?,?)", 1, 1, 1, 1,datetime.today(),'2')
#connection.commit()

print(df.shape)
```

```
df.drop_duplicates(inplace=True)
print(df.shape)


# # read in features file

# In[9]:

FEATURES_PATH = os.path.join(dir_to_save_files, 'EHR_MAIN_FEATURES.csv')
features_df = pd.read_csv(FEATURES_PATH)


features_dict = {name:list(col.dropna()) for name,col in features_df.items()}
print(features_dict.keys())

final_features = features_dict['final_features']
cat_to_drop = features_dict['cat_to_drop']
outcome_vars = features_dict['outcome_vars']
feat_to_drop = features_dict['feat_to_drop']
cat_vars = features_dict['cat_vars']
contin_vars = features_dict['contin_vars']
bool_outcome_vars = features_dict['bool_outcome_vars']

# In[10]:


df[contin_vars] = df[contin_vars].astype(float)
df[cat_vars] = df[cat_vars].astype(object)
#df["ASA_STATUS"] = df["ASA_STATUS"].astype(float)

# remove HRS_ADMSN_TO_SURGERY from feature list
try:
    final_features.remove('HRS_ADMSN_TO_SURGERY')
except ValueError:
    pass

if 'HRS_ADMSN_TO_SURGERY' not in feat_to_drop:
    feat_to_drop = feat_to_drop.append('HRS_ADMSN_TO_SURGERY')

#cursor.execute("INSERT INTO PerioperativeDM.SCRATCH.Mortality_Prediction VALUES
(?,?,?,?,?,?)", 1, 1, 1, 1,datetime.today(),'3')
#connection.commit()
# # Get acceptable values for categorical variables and filter

# In[19]:
```

```
def read_acceptable_vals(filename):
    with open(os.path.join(data_dir, filename)) as f:
        return [l.strip() for l in f.readlines()]

pre_surg_location_vals = read_acceptable_vals("PRE_SURG_LOCATION_unique_values.txt")
pat_class_vals = read_acceptable_vals("PAT_CLASS_unique_values.txt")
hcup_code_vals = read_acceptable_vals("HCUP_CODE_unique_values.txt")
gender_vals = read_acceptable_vals("GENDER_unique_values.txt")
case_srv_name_vals = read_acceptable_vals("CASE_SRV_NAME_unique_values.txt")


# In[20]:

print (pat_class_vals)
print(case_srv_name_vals)


# In[21]:


#df = df[df.PRE_SURG_LOCATION.isin(pre_surg_location_vals)]
print(df.shape)
df = df[df.GENDER.isin(gender_vals)]
print(df.shape)
df = df[df.PAT_CLASS.isin(pat_class_vals)]
print(df.shape)
#df = df[df.HCUP_CODE.astype(float).isin(hcup_code_vals)]
##print(df.shape)
df = df[df.GENDER.isin(gender_vals)]
print(df.shape)
df = df[df.CASE_SRV_NAME.isin(case_srv_name_vals)]
print(df.shape)
print(df)
#cursor.execute("INSERT INTO PerioperativeDM.SCRATCH.Mortality_Prediction VALUES
(?,?,?,?,?,?)", 1, 1, 1, 1,datetime.today(),'3.2')
#connection.commit()

# # Filter out surgeries that don't occur in RR or SM operating rooms

# In[22]:


#print(df.LOCATION_GROUP.unique())
#df = df[df['LOCATION_GROUP'].isin(['RR OR', 'SM OR','SM SC','SM OB OR','RR OB
OR'])]
```

```
print(df.shape)
#df = df[df['LOCATION_GROUP'].isin(['RR OR', 'SM OR'])]
print(df.shape)


# # Filter out surgeries that were not INPATIENT, SAME DAY ADMIT, EMERGENCY, or
OVERNIGHT RECOVERY

# In[23]:


print("Shape before filtering out outpatient surgeries:", df.shape)
#df = df[df['PATIENT_CLASS'].isin(['INPATIENT', 'SAME DAY ADMIT', 'EMERGENCY',
'OVERNIGHT RECOVERY'])]
df = df[df['PAT_CLASS'].isin(['INPATIENT', 'SAME DAY ADMIT',
'EMERGENCY','SURGERY OUTPATIENT' ,'OVERNIGHT RECOVERY'])]
print("Shape after filtering out outpatient surgeries:", df.shape)
#cursor.execute("INSERT INTO PerioperativeDM.SCRATCH.Mortality_Prediction VALUES
(?,?,?,?,?,?)", 1, 1, 1, 1,datetime.today(),'3.3')
#connection.commit()

# # Filter based on ASA status, age

# In[24]:


'''
try:
    print("Shape before filtering out based on ASA_STATUS:", df.shape)
    print("ASA_STATUS mean:", df.ASA_STATUS.mean())
    df = df[(df["ASA_STATUS"] <= MAX_ASA_STATUS) & (df["ASA_STATUS"] >=
MIN_ASA_STATUS)]
    print("Shape after filtering out based on ASA_STATUS:", df.shape)
    print("ASA_STATUS mean:", df.ASA_STATUS.mean())
except AttributeError:
    pass
print("==================================")
print("Mean age:", df.AGE_LT_90.mean())
print("STD age:", df.AGE_LT_90.std())
df = df[(df["AGE_LT_90"] <= MAX_AGE) & (df["AGE_LT_90"] >= MIN_AGE)]
print("Mean age:", df.AGE_LT_90.mean())
print("STD age:", df.AGE_LT_90.std())
print("Shape after filtering out based on AGE_LT_90:", df.shape)
'''
```

```
#cursor.execute("INSERT INTO PerioperativeDM.SCRATCH.Mortality_Prediction VALUES
(?,?,?,?,?,?)", 1, 1, 1, 1,datetime.today(),'3.4')
#connection.commit()

# # check demographic distributions

# In[25]:


# print "Number of Patients:", df.shape[0]
# print "Patients with in-hospital mortality: {}
({}%)".format(df.INPT_DEATH_YN.value_counts()[1],
df.INPT_DEATH_YN.value_counts(normalize="True")[1]*100)
# print "Mean age:", df.AGE_LT_90.mean(), " std:", df.AGE_LT_90.std()
print("Number of female patients: {} ({}%)".format(df[df["GENDER"] == "F"].shape[0],
df[df["GENDER"] == "F"].shape[0]/float(df.shape[0])*100))

# try:
#     print "Number of patients in RR OR: {}
({}%)".format(df.LOCATION_GROUP.value_counts()["RR OR"],
df.LOCATION_GROUP.value_counts(normalize="True")["RR OR"]*100)
#     print "Number of patients in SM OR: {}
({}%)".format(df.LOCATION_GROUP.value_counts()["SM OR"],
df.LOCATION_GROUP.value_counts(normalize="True")["SM OR"]*100)
#     #print "Number of patients in SM SC: {}
({}%)".format(df.LOCATION_GROUP.value_counts()["SM SC"],
df.LOCATION_GROUP.value_counts(normalize="True")["SM SC"]*100)
# except AttributeError:
#     pass
# print("="*40)
# try:
#     print "ASA Status:", df.ASA_STATUS.value_counts()
#     print "ASA Status (%):", (df.ASA_STATUS.value_counts()/df.shape[0])*100
# except AttributeError:
#     pass

# print("="*40)
# print "Mean age of mortalities:", df[df["INPT_DEATH_YN"] == 1].AGE_LT_90.mean(), "
std:", df[df["INPT_DEATH_YN"] == 1].AGE_LT_90.std()
# print("Number of female mortalities: {} ({}%)".format(df[df["GENDER"] ==
"F"]["INPT_DEATH_YN"].sum(),
#                                                      df[df["GENDER"] ==
"F"]["INPT_DEATH_YN"].sum()/float(df["INPT_DEATH_YN"].sum())*100))
# print("Number of male mortalities: {} ({}%)".format(df[df["GENDER"] ==
"M"]["INPT_DEATH_YN"].sum(),
```

```
#                                        df[df["GENDER"] ==
"M"]["INPT_DEATH_YN"].sum()/float(df["INPT_DEATH_YN"].sum())*100))
# try:
#     print("="*40)
#     print("Number of mortalities stratified by location")
#     print(df.groupby("LOCATION_GROUP")["INPT_DEATH_YN"].sum())
#
print(df.groupby("LOCATION_GROUP")["INPT_DEATH_YN"].sum()/float(df["INPT_DEAT
H_YN"].sum())*100)
# except AttributeError:
#     pass
# print("="*40)
# print("Number of mortalities stratified by ASA status")
# print(df.groupby("ASA_STATUS")["INPT_DEATH_YN"].sum())
#
print(df.groupby("ASA_STATUS")["INPT_DEATH_YN"].sum()/float(df["INPT_DEATH_YN"
].sum())*100)

# if verbose:
#     print(df["CASE_SRV_NAME"].value_counts())
#     print(df["CASE_SRV_NAME"].value_counts()/df.shape[0])*100
#     print("="*40)
#     print(df[df["INPT_DEATH_YN"] == 1]["CASE_SRV_NAME"].value_counts())
#     print(df[df["INPT_DEATH_YN"] ==
1]["CASE_SRV_NAME"].value_counts()/df["INPT_DEATH_YN"].sum())*100

#cursor.execute("INSERT INTO PerioperativeDM.SCRATCH.Mortality_Prediction VALUES
(?,?,?,?,?,?)", 1, 1, 1, 1,datetime.today(),'5')
#connection.commit()


# # Remove outlier values

# In[26]:



# string_cols = ['PRE_SURG_LOCATION', 'CASE_SRV_NAME_GROUP',
'CASE_SRV_NAME', 'PRIMARY_CPT',
#                          'GENDER', 'HCUP_DESC', 'CPT_DESC', 'PAT_CLASS',
'OR_CASE_ID', 'ADMSN_ID']
# dff = df.drop(['PRE_SURG_LOCATION', 'CASE_SRV_NAME_GROUP',
'CASE_SRV_NAME', 'PRIMARY_CPT',
#                          'GENDER', 'HCUP_DESC', 'CPT_DESC', 'PAT_CLASS',
'OR_CASE_ID', 'ADMSN_ID'], axis=1)
# #dff = df.select_dtypes(include=['float64'])
# dff = df[contin_vars]
```

```
# df_string_cols = df[df.columns.difference(dff.columns.values)]
# print df_string_cols.columns.values
# #display(dff.describe())
# print (np.abs(st.zscore(dff, axis=1)) > 3)
# #print dff.sub(dff.mean()).div(dff.std()).abs().lt(3)
# df_no_outliers = dff[dff.sub(dff.mean()).div(dff.std()).abs().lt(4)]
# df_no_outliers[df_string_cols.columns.values] = df_string_cols
# if verbose:
#     display(df_no_outliers.describe(include="all"))
# df = df_no_outliers


# # Remove variables related to lab times (i.e. *.HRS_2_SURGERY)

# In[27]:


#remove variables that have to do with time
# cols_to_keep_no_hrs2surgery = [c for c in df.columns if not
c.endswith(".HRS_2_SURGERY")]
# print cols_to_keep_no_hrs2surgery
# print len(cols_to_keep_no_hrs2surgery)
# df=df[cols_to_keep_no_hrs2surgery]

#cursor.execute("INSERT INTO PerioperativeDM.SCRATCH.Mortality_Prediction VALUES
(?,?,?,?,?,?)", 1, 1, 1, 1,datetime.today(),'6')
#connection.commit()
# # Remove unnecessary features

# In[28]:


# save this for checking predictions over time
#admsn_surgery_number = df["ADMSN_SURGERY_NUMBER"]
#print(admsn_surgery_number.shape)
or_case_id_number = df["OR_CASE_ID"]
PAT_CLASS_PR= df["PAT_CLASS"]
#admsn_ids = df['ADMSN_ID']


# In[29]:


df = df[final_features + [TARGET_VARIABLE]]
```

```
# # One-hot encode categorical variables

# In[30]:


for var in cat_vars:
    try:
        # drop_first uses k-1 dummies out of k categories
        print(var)
        #df = pd.get_dummies(df, columns=[var], drop_first=True)
        df = pd.get_dummies(df, columns=[var])
        pass
    except ValueError:
        pass
    except KeyError:
        print(var, 'already dropped')
# remove categorical variables (string values)
for var in cat_vars:
    try:
        df.drop(var, axis=1, inplace=True)
        pass
    except ValueError:
        print(var, 'already dropped')
    except KeyError:
        print(var, 'already dropped')


# # Remove features we don't want to include

# In[31]:


# if len(feature_whitelist) == 0:
for cat in cat_to_drop:
    try:
        df.drop(cat, axis=1, inplace=True)
    except KeyError:
        print(cat, 'already dropped')
#print df.columns.values
for col in sorted(df.columns.values):
    print(col, "\t\t", df[col].dtype)


# # Remove target variables from data frame

# In[32]:
```

```
print(df[TARGET_VARIABLE].mean())
print("Column names:", df.columns.values)
try:
    y = np.ravel(df[TARGET_VARIABLE])
    #asa_status = df["ASA_STATUS"]
    df.drop(TARGET_VARIABLE, axis=1, inplace=True, errors='ignore')
    df.drop(outcome_vars, axis=1, inplace=True, errors='ignore')
    input_death_yn = df['INPT_DEATH_YN']
except KeyError:
    print(TARGET_VARIABLE, "already dropped")
# http://scikit-learn.org/stable/modules/generated/sklearn.preprocessing.Imputer.html
# default stragegy: mean
# if len(feature_whitelist) > 0:
#     feature_whitelist = [c for c in feature_whitelist if not c.endswith(".HRS_2_SURGERY")]
#     df = df[feature_whitelist]
print(df.isnull().sum())
print(y.sum())

#cursor.execute("INSERT INTO PerioperativeDM.SCRATCH.Mortality_Prediction VALUES
(?,?,?,?,?,?)", 1, 1, 1, 1,datetime.today(),'7')
#connection.commit()
# # Make sure we have all features that we used to train model

# In[33]:


final_feature_list = pd.read_csv(os.path.join(dir_to_save_files, exp_prefix +
"_final_feature_list.txt"), header=None)
print(final_feature_list.shape)


# In[34]:


for col in final_feature_list[0]:
    if col not in df.columns.values:
        print(col, "missing from dataframe")
        df[col] = np.nan


df.columns.values

df = df[final_feature_list[0]]
```

```
df.columns.values


# # Standardize training, testing data

class StandardizeWithNaN(TransformerMixin, BaseEstimator):
    '''This estimator is for standardizing a dataset that has missing data'''
    def __init__(self):
        self.X_mean = []
        self.X_std = []
        pass

    def fit(self, X, y=None):
        # get mean and standard deviation of columns
        self.X_mean = np.nanmean(X, axis=0)
        self.X_std  = np.nanstd(X, axis=0)
        return self

    def transform(self, X):
        # subtract mean and divide by standard deviation
        return (X - self.X_mean)/self.X_std


# In[40]:

#cursor.execute("INSERT INTO PerioperativeDM.SCRATCH.Mortality_Prediction VALUES
(?,?,?,?,?,?)", 1, 1, 1, 1,datetime.today(),'8')
#connection.commit()

#scaler = StandardizeWithNaN()
# scaler.fit(X_test)
# X_test = scaler.transform(X_test)
#scaler.fit(df)
scaler = pickle.load(open(os.path.join(model_dir, "StandardizeWithNaN.pkl"), "rb"))
X_test = scaler.transform(df)
y_test = y


# # Impute missing values

# In[41]:


class SoftImputeEstimator(TransformerMixin, BaseEstimator):
    '''This estimator is for wrapping the SoftImpute algorithm'''
    def __init__(self, max_iters=200, verbose=True):
```

```
        self.max_iters = max_iters
        self.verbose = verbose
        self.fit_count = 0
        self.transform_count = 0
        pass

    def fit(self, X, y=None):
        self.fit_count += 1
        print("SoftImputeEstimator fit count: {}".format(self.fit_count))
        return self

    def transform(self, X):
        self.transform_count += 1
        print("SoftImputeEstimator transform count: {}".format(self.transform_count))
        try:
            # subtract mean and divide by standard deviation
            return SoftImpute(max_iters=self.max_iters,
verbose=self.verbose).complete(X.replace(np.inf, np.nan))
        # ValueError raised if no values need to be imputed
        except ValueError:
            return np.array(X)


# In[42]:


print("imputing X_test")
#print(np.isnan(X_test).any())
#si = SoftImputeEstimator()
si = pickle.load(open(os.path.join(model_dir, "MeanImputer.pkl"), "rb"))
si.statistics_[np.isnan(si.statistics_)] = 0.
print(si.statistics_)
X_test = si.transform(X_test.replace(np.inf, np.nan))


# # Load model(s)

# In[43]:


# models = {}
models = []
for i in range(1):
    model_file_name = "Random Forest_train_sk{}.pkl".format(sk_version)
    model = pickle.load(open(os.path.join(model_dir, model_file_name), "rb"), encoding='latin1')
    print("Loaded", model_file_name)
```

```
    #models["fold_{}".format(i)] = model
    models.append(model)


# In[44]:


models[0]


# # Predict classes and get probability of labels

# In[45]:


# model_predictions = {k: model.predict(np.array(X_test)) for k, model in models.items()}
# model_probs = {k: model.predict_proba(X_test) for k, model in models.items()}

#model_predictions = [model.predict(np.array(X_test)) for model in models]
#model_probs = [model.predict_proba(X_test) for model in models]


# predict class labels for the test set
def predict_given_threshold(probs, threshold=0.15):
    return [True if x[1] > threshold else False for x in probs]

# get probability of mortality from model
model_probs = [model.predict_proba(X_test) for model in models]
# any probability over the threshold is predicted True, else False
model_predictions = [predict_given_threshold(probs, threshold=0.5) for probs in model_probs]


#cursor.execute("INSERT INTO PerioperativeDM.SCRATCH.Mortality_Prediction VALUES
(?,?,?,?,?,?)", 1, 1, 1, 1,datetime.today(),'8.1')
#connection.commit()




model_names = ["Random Forest"]


###cursor.execute("INSERT INTO PerioperativeDM.SCRATCH.Mortality_Prediction
VALUES (?,?,?,?,?,?)", 1, 1, 1, 1,datetime.today(),'9')
###connection.commit()
```

```
#if verbose:
#    for i in zip(model_probs[0][:,1], or_case_id_number, y):
#        print(i)


#cursor.execute("INSERT INTO PerioperativeDM.SCRATCH.Mortality_Prediction VALUES
(?,?,?,?,?,?)", 1, 1, 1, 1,datetime.today(),'9.1')
#connection.commit()

#plt.hist(model_probs[0][y == False,1], bins=20)
#plt.hist(model_probs[0][y == True,1], bins=20)
#plt.xlabel("Probability")
#plt.ylabel("Patient Count")
#plt.show()

#cursor.execute("INSERT INTO PerioperativeDM.SCRATCH.Mortality_Prediction VALUES
(?,?,?,?,?,?)", 1, 1, 1, 1,datetime.today(),'9.2')
#connection.commit()

# In[55]:


#print(model_probs[0][y == False,1].mean())
#print(model_probs[0][y == False,1].std())
#print(model_probs[0][y == True,1].mean())
#print(model_probs[0][y == True,1].std())


# In[56]:


#cursor.execute("INSERT INTO PerioperativeDM.SCRATCH.Mortality_Prediction VALUES
(?,?,?,?,?,?)", 1, 1, 1, 1,datetime.today(),'112')
#connection.commit()

#cursor = connection.cursor()
def bool_to_int(x):
    if x==True:
        return 1
    else:
        return 0
for i in zip(or_case_id_number, model_probs[0][:,1], y,
model_predictions[0],PAT_CLASS_PR):
    print(i[0], i[1], bool_to_int(i[2]), bool_to_int(i[3]))
    cursor.execute("INSERT INTO PerioperativeDM.SCRATCH.Mortality_Prediction VALUES
(?,?,?,?,?,?)", i[0], i[1], bool_to_int(i[2]), bool_to_int(i[3]),datetime.today(),i[4])
```

```
    connection.commit()
cursor.close()
connection.close()

# In[ ]:
```
